# BMC *Endocrine Disorders* reviewer acknowledgement 2014

**DOI:** 10.1186/1472-6823-15-3

**Published:** 2015-02-12

**Authors:** Tim Shipley

**Affiliations:** BioMed Central, Floor 6, 236 Gray’s Inn Road, London, WC1X 8HB UK

## Abstract

The editors of BMC Endocrine Disorders would like to thank all our reviewers who have contributed to the journal in Volume 14 (2014).

## Reviewer acknowledgement

**Zbigniew Adamczewski**

Poland

**Amar Agha**

Ireland

**Nk Agrawal**

India

**Manuela Albertelli**

Italy

**Theresa Allain**

Malawi

**Madson Almeida**

Brazil

**Alireza Amirbaigloo**

Iran

**Decio Armanini**

Italy

**Victor M Baizabal-Aguirre**

Mexico

**Wei Bao**

United States of America

**Ilaria Barchetta**

Italy

**Marisa Bare**

Spain

**Katharine Barnard**

United Kingdom

**Analabha Basu**

India

**Nicholas Beare**

United Kingdom

**Giorgio Bedogni**

Italy

**Lucy Ann Behan**

Canada

**Amaya Belanger-Quintana**

Spain

**Abdulbari Bener**

Qatar

**Katrien Benhalima**

Belgium

**David Beran**

Switzerland

**Elizabeth Beverly**

United States of America

**Peyman Björklund**

Sweden

**Kristien Boelaert**

United Kingdom

**Marek Bolanowski**

Poland

**Michael Bonkowski**

United States of America

**Jérémie Botton**

France

**Barbara J Boucher**

United Kingdom

**Jacqueline Boyle**

Australia

**Monika Buraczynska**

Poland

**Michael Burch United**

Kingdom

**Marie-José Butel**

France

**Atilla Buyukgebiz**

Turkey

**Milena Caldato**

Brazil

**Adrian Cameron**

Australia

**Alessandra Care'**

Italy

**Manuel Castro Cabezas**

Netherlands

**Filomena Cetani**

Italy

**Giriraj Chandak**

India

**Yi-Cheng Chang**

Taiwan

**Yuan-Jia Chen**

China

**Shih-Ping Cheng**

Taiwan

**Ching-Ju Chiu**

Taiwan

**Gabriel Chodick**

Israel

**Mary Chong**

Singapore

**Dirk Lund Christensen**

Denmark

**Pm Clark United**

Kingdom

**Angela Clow**

United States of America

**Neale Cohen**

Australia

**Bernard Conte-Devolx**

France

**Flavia Costa- Barbosa**

Brazil

**Rachel Crowley**

Ireland

**Alberto Da Silva Moraes**

Brazil

**Eleonora Dalla Bella**

Italy

**Shannon Davis**

United States of America

**Giovambattista Desideri**

Italy

**Thomas Dickmeis**

Germany

**George Dimitriadis**

Greece

**Sima Djalali**

Switzerland

**Horacio Domené**

Argentina

**Periklis Dousdampanis**

Greece

**Koen Dreijerink**

Netherlands

**Juan Duenas**

Colombia

**Carlos S Duque**

Colombia

**Peter Ebeling**

Australia

**Roberto Esposito**

Italy

**Denis Evoy**

Ireland

**Oana Falup-Pecurariu**

Romania

**Fabio Faucz**

United States of America

**E. Vincent Faustino**

United States of America

**Huai Feng**

United States of America

**Fabio Fernandes-Rosa**

France

**Bruno Ferraz-De-Souza**

Brazil

**Charles Fox United**

Kingdom

**Chris Frampton New**

Zealand

**Davide Francomano**

Italy

**Yoshio Fujioka**

Japan

**Filip Gabalec**

Czech Republic

**Roger Gadsby**

United Kingdom

**Claudia Gagnon**

Canada

**Neetika Garg**

United States of America

**Erica Gentilin**

Italy

**Peter Gergics**

United States of America

**Laura Gianotti**

Italy

**Oliver Gimm**

Sweden

**Paul Glynn**

United Kingdom

**Mukesh Gohel**

India

**Cumali Gokce**

Turkey

**Larissa Gomes**

Brazil

**Elise Gomez-Sanchez**

United States of America

**Margaret Grey**

United States of America

**Maria Rosaria Gualano**

Italy

**Martin Gulliford**

United Kingdom

**Vipin Gupta**

India

**Rubina Hakeem**

Pakistan

**Thang Han**

United Kingdom

**Nihal Hatipoglu**

Turkey

**Jan Willem Haveman**

Netherlands

**Weimin He**

China

**Ian Holdaway**

New Zealand

**Deborah Jane Holmes-Walker**

Australia

**Fei-Hsiu Hsiao**

Taiwan

**Peter Igaz**

Hungary

**Richard Ijzerman**

Netherlands

**Romaina Iqbal**

Pakistan

**Somchit Jaruratanasirikul**

Thailand

**Michelle Jones**

United States of America

**Prapaporn Jungtrakoon**

United States of America

**Gregory Kaltsas**

Greece

**Zuleyha Karaca**

Turkey

**Anuradhani Kasturiratne**

Sri Lanka

**Prasad Katulanda**

Sri Lanka

**Atsunari Kawashima**

Canada

**Fahrettin Kelestimur**

Turkey

**Marcus Kennedy**

Ireland

**Anuradha Khadilkar**

India

**Muhammad Kibriya**

United States of America

**Dong-Jun Kim**

Korea, South

**Christian Koch**

United States of America

**Josef Köhrle**

Germany

**Alexander Kokkinos**

Greece

**Michiharu Komatsu**

Japan

**Evangelos Kontopantelis**

United Kingdom

**Juraj Koska**

United States of America

**Nermin Kösüs**

Turkey

**James Krinsley**

United States of America

**Grace Marie Ku**

Philippines

**Ganesh Kumar**

United States of America

**Tommy Kyaw-Tun**

Ireland

**George Kyriazis**

United States of America

**Stefano La Rosa**

Italy

**Pauline Siew Mei Lai**

Malaysia

**Vaia Lambadiari**

Greece

**Lisa Langsetmo**

Canada

**Mary Larkin**

United States of America

**Anna Lauber-Biason**

Switzerland

**David Lebeaux**

France

**Duk-Hee Lee**

Korea, South

**Antonio Lerario**

Brazil

**Jianhong Li**

China

**Xiaoying Li**

Canada

**Stavros Liatis**

Greece

**Rossella Libe**

France

**Suh-Jen Lin**

United States of America

**Yijia Lou**

Comoros

**Paul Lucassen**

Netherlands

**Naim Maalouf**

United States of America

**Sujay Madduri**

United States of America

**Dianna Magliano**

Australia

**Michele Malaguarnera**

Italy

**Rayaz Malik**

United Kingdom

**Ashwini Mallappa**

United States of America

**Robert Mallet**

United States of America

**Jessica Markowitz**

United States of America

**Vincenzo Marotta**

Italy

**Raman Marwaha**

India

**Melinda Maryniuk**

United States of America

**Paolo Marzullo**

Italy

**Rocio Mateo-Gallego**

Spain

**Sandeep K Mathur**

India

**Gherardo Mazziotti**

Italy

**Ciara Mcdonnell**

Ireland

**Malachi Mckenna**

Ireland

**Mark Mclean**

Australia

**Diane Mege**

France

**Nicholas Mezitis**

United States of America

**Teresa Mezza**

Italy

**Paras Kumar Mishra**

United States of America

**Marie Misso**

Australia

**Maureen Monaghan**

United States of America

**Carla Moran**

United Kingdom

**Yuji Moriwaki**

Japan

**Thomas Mueller**

Austria

**Arijit Mukhopadhyay**

India

**Giovanna Muscogiuri**

Italy

**Parimala Narne**

India

**Michael Nauck**

Germany

**Juan Nicola**

Argentina

**Michael O'Reilly**

United Kingdom

**Zeynep Osar Siva**

Turkey

**Tríona O'Shea**

Ireland

**Nikolaos Papanas**

Greece

**Renato Pasquali**

Italy

**Klara Paudel**

United Kingdom

**Andre Peinnequin**

France

**Phillip Pekala**

United States of America

**M. Yanina Pepino**

United States of America

**Luigi Petramala**

Italy

**Elzbieta Petriczko**

Poland

**Daniel Petrovic**

Slovenia

**Gustavo Duarte Pimentel**

Brazil

**Chang Ping'An**

China

**Milan Piya**

United Kingdom

**Giuseppe Procopio**

Italy

**Manuel Puig-Domingo**

Spain

**Fanny Rancière**

France

**Jonas Ranstam**

Sweden

**Ulrich Renner**

Germany

**Musarrat Riaz**

Pakistan

**Evangelos Rizos**

Greece

**Karine Rizzoti**

United Kingdom

**Jo-Anne Rizzotto**

United States of America

**Edna Roche**

Ireland

**Vincenzo Rochira**

Italy

**Santiago Rodriguez-Segade**

Spain

**William Rojas**

Colombia

**Vanessa Ronconi**

Italy

**Gian Paolo Rossi**

Italy

**Mario Rotondi**

Italy

**Marek Ruchala**

Poland

**R.A.C. Ruiter**

Netherlands

**Isabelle Runkle De La Vega**

Spain

**Banshi Saboo**

India

**Matteo Santoni**

Italy

**Vinod Scaria**

India

**Carlos Alberto Scrideli**

Brazil

**Teresa Maria Seccia**

Italy

**Alissa Segal**

United States of America

**Yutaka Seino**

Japan

**Arundhati Sharma**

India

**Mark Sherlock**

Ireland

**Cephas Sialubanje**

Zambia

**Diarmuid Smith**

Ireland

**Miguel A Sogorb**

Spain

**Martin Soubrier**

France

**Carole Spencer**

United States of America

**Seamus Sreenan**

Ireland

**Tobias Stalder**

Germany

**Douglas Steinke**

United Kingdom

**Jan Stepan**

Czech Republic

**Joanna Stewart**

New Zealand

**Monique Stone**

Australia

**Michael Stowasser**

Australia

**Alexios Strimpakos**

Greece

**Elsa Strotmeyer**

United States of America

**Gonca Tamer**

Turkey

**Nagaaki Tanaka**

Japan

**Molly Tanenbaum**

United States of America

**Francesco Tassone**

Italy

**Nicholas Tentolouris**

Greece

**Ajay Thankamony**

United Kingdom

**Jerome Thevenot**

Finland

**Sathish Thirunavukkarasu**

Australia

**Debbie Thompson**

Jamaica

**Rodrigo Toledo**

United States of America

**Jeremy Tomlinson**

United Kingdom

**Jorg Tost**

France

**Ericka Trarbach**

Brazil

**Panagiotis Tsapogas**

Greece

**Toshihiko Tsukada**

Japan

**Tiinamaija Tuomi**

Finland

**Kultigin Turkmen**

Turkey

**Mariko Uehara**

Japan

**Zoltan Ungvari**

United States of America

**Vytautas Usonis**

Lithuania

**Mukremin Uysal**

Turkey

**Tone Gretland Valderhaug**

Norway

**Katherine Valeros**

Singapore

**André Van Beek**

Netherlands

**Danielle Van Der Kaay**

Canada

**Aj Van Der Lely**

Netherlands

**Tom Van Herpe**

Belgium

**Emerielle Vanzela**

Brazil

**Dimitra Argyro Vassiliadi**

Greece

**Srividya Vasu**

United Kingdom

**Alejandro Velez Hoyos**

Colombia

**Kavita Venkataraman**

Singapore

**Giuseppe Vezzoli**

Italy

**Mary Vouyiouklis**

United States of America

**Xiangbing Wang**

United States of America

**Susan Webb**

Spain

**Jan-Maarten Wit**

Netherlands

**Viroj Wiwanitkit**

Thailand

**Wenxun Wu**

China

**Yuichiro Yamada**

Japan

**Kazuki Yasuda**

Japan

**Xingwang Ye**

China

**Ozcan Yildiz**

Turkey

**Danxia Yu**

United States of America

**Carles Zafon**

Spain

**Raul Zamora Ros**

France

**Yan Zheng**

United States of America

